# Population Pharmacodynamic Analyses of Human Anti-Rabies Virus Monoclonal Antibody (Ormutivimab) in Healthy Adult Subjects

**DOI:** 10.3390/vaccines10081218

**Published:** 2022-07-29

**Authors:** Junnan Zhang, Nianmin Shi, Guohua Li, Li Li, Yunhua Bai, Liqing Yang, Weimin Zhao, Jian Gao, Jingshuang Wei, Wei Zhao, Lili Zhai, Peiyuan Huo, Lemin Ren, Lan Yu, Yufeng Li

**Affiliations:** 1 Center for Disease Control and Prevention of Chaoyang District, Beijing 100021, China; zjn406822346@126.com (J.Z.); shouyilili@163.com (L.L.); 13651367636@126.com (Y.B.); cycdpc2003@163.com (L.Y.); 2 Beijing Institute of Biological Products, Beijing 100021, China; 13911215549@163.com; 3 Centre for Disease Control and Prevention of Shanxi Provincial, Taiyuan 030012, China; lgh13934530294@163.com (G.L.); kkb105@163.com (W.Z.); 4 NCPC New Drug Research and Development Co., Ltd., State Key Laboratory of Antibody Research & Development, Shijiazhuang 050015, China; gaojian2989@163.com (J.G.); weijsh1979@163.com (J.W.); 13231183627@163.com (W.Z.); lili_zhai@163.com (L.Z.); 15903213467@163.com (P.H.); 13171800169@163.com (L.R.); yulan2680@163.com (L.Y.)

**Keywords:** Ormutivimab, NONMEM, rabies, monoclonal antibody, rabies vaccine

## Abstract

Ormutivimab is the first recombinant human anti-rabies monoclonal antibody (rhRIG) approved for clinical application in China. In this study, a population pharmacodynamic (PPD) model was established to compare the neutralizing antibody activities of Ormutivimab and human rabies immunoglobulin (HRIG), alone or combined with human rabies vaccine (Vero), in a phase II clinical trial, and to recommend a target dose for the phase III trial. The model was verified to fit the PPD data well. The stability of the model was verified by the bootstrap method. The level of neutralizing antibodies in vivo increased rapidly after administration of Ormutivimab or HRIG. Neutralizing antibodies with a strong activity were produced at 7 days (Ormutivimab + vaccine) or 10 days (HRIG + vaccine) after induction by the vaccine in vivo. Compared to that induced by HRIG + vaccine, the level of the neutralizing antibodies induced by Ormutivimab + vaccine peaked higher and faster. The levels of neutralizing antibodies induced by Ormutivimab + vaccine and HRIG + vaccine were similar within 21 days after administration. According to these results and the safety data, 20 IU·kg^−1^ was recommended as the target dose in the confirmatory study of Ormutivimab. Registration: ClinicalTrials.gov #NCT02559921.

## 1. Introduction

Rabies is estimated to cause over 59,000 human deaths worldwide each year, mostly in rural areas of developing countries, and has been listed as a neglected tropical disease by the WHO [1]. Timely administration of immunoglobulin helps prevent rabies during post-exposure prophylaxis (PEP). Regardless of the source of viral exposure, human rabies is preventable with proper wound care and prompt administration of vaccine and rabies immune globulin (RIG). Traditional RIGs used for human rabies PEP are polyclonal immunoglobulins, derived either from the plasma of immunized human donors, human rabies immune globulin (HRIG), or from animals, such as horses (equine rabies immunoglobulin, ERIG) [2]. Despite its high effectiveness, HRIG has several drawbacks, such as the insufficient supply in endemic areas, batch-to-batch variations, relatively high costs, and safety concerns due to its blood-derived nature. Hence, new products are needed to replace the plasma-derived preparations in the PEP treatment of rabies [3], and recombinant monoclonal antibodies are prospective substitutes [4].

At present, only three recombinant human anti-rabies monoclonal antibodies (rhRIG) have been approved for clinical application globally. SIIRMab (Rabishield) by the Serum Institute of India PVT. LTD. (SIIPL), which was licensed in 2016 and launched in 2017 in India [5], is a single human IgG1 type mAb that binds to a conformational epitope of the rabies glycoprotein. The mAb was originally derived from transgenic mice and developed by MassBiologics (Boston, MA, USA), under the name 17C7, before being transferred to SIIPL for further development, under the name SII RMAb [6]. Another product, RabiMabs, which was developed by the Zydus Cadila Research Center in India and approved for marketing in India in September 2019, is a cocktail of two mAbs targeting different non-overlapping epitopes.

Ormutivimab, the first recombinant human anti-rabies monoclonal antibody in China, was produced by Chinese hamster ovary (CHO) cells and launched in 2022. Its ability to neutralize a variety of rabies viruses has been validated via in vitro and in vivo assays [7]. Research institutes in China have also proven that Ormutivimab can neutralize a broad panel of Chinese prevalent street rabies viruses. The selected strains are isolated from a variety of hosts, including dogs, bats, deer, and Chinese ferret-badgers in endemic regions in China and are involved in all the main branches of the phylogenetic tree. The results of the phase I trial have showed that Ormutivimab is comparable or superior to HRIG in its safety and neutralizing activity, and suggested a dose range of 20 IU·kg^−1^ to 40 IU·kg^−1^ for further study and evaluation [8].

Therefore, we conducted a phase II study to preliminarily evaluate the safety and rabies virus neutralizing antibody (RVNA) activity of two doses of Ormutivimab (20 IU·kg^−1^ and 40 IU·kg^−1^) in healthy adults, comparing them with those of HRIG. This article characterized the population pharmacodynamic (PPD) properties of different doses of Ormutivimab in healthy adult subjects.

## 2. Participants and Methods

### 2.1. Clinical Study

This was a randomized, double-blind, parallel-group phase II clinical study (ClinicalTrials.gov Identifier: NCT02559921). The study protocol was designed and performed according to the Declaration of Helsinki and the International Conference Harmonization Guidelines for Good Clinical Practice. Prior to study initiation, the protocol, informed consent form, and other study-related documents were approved by the ethics committee of the CDC, Chaoyang District, Beijing, and the CDC, Shanxi.

Healthy adults, aged 18 to 55, without a history of rabies exposure or immunization (rabies vaccine and/or immunoglobulin injection) were eligible in the study. The exclusion criteria included fever higher than 37 °C (axillary); acute disease or infection; immune diseases; treatment with immunosuppressive or immune enhancer drugs; use of hormone drugs; allergy to any vaccine, blood derived products, or antibodies; other immunization previously scheduled; pregnancy, planned pregnancy or breastfeeding during the study; mental, heart, liver, kidney, blood, and other organ system diseases or functional disorders; plasma donation (within 7 days); massive blood loss (within 56 days); blood or blood products transfusion (within 6 months); and other conditions not compatible with the study protocol. Written informed consents were obtained from all volunteers before being screened.

The total number of subjects was 300 (60 in phase IIa and 240 in phase IIb). The 60 subjects in the phase IIa trial were randomly assigned to 3 groups. The subjects in each group received Ormutivimab (20 IU·kg^−1^) only, Ormutivimab (40 IU·kg^−1^) only, or HRIG (20 IU·kg^−1^) only. The subjects in the phase IIb trial were randomly assigned to 4 groups. The subjects in each group received Ormutivimab (20 IU·kg^−1^), Ormutivimab (40 IU·kg^−1^), HRIG (20 IU·kg^−1^), or placebo, in combination with rabies vaccine.

### 2.2. Drugs

Ormutivimab (200 IU/1 mL/vial, Lot No. 20131003; or 500 IU/2.5 mL/vial, Lot No. 20131004) and the excipient (2.5 mL/vial, Lot No. 20131002) were manufactured by North China Pharmaceutical Co Ltd., Shijiazhuang, China. HRIG (200 IU/vial/2 mL, Lot No. 20130714, titer 100 IU/mL) was manufactured by Shuanglin Biopharmaceutical Co., Ltd., Guangdong, China. The human rabies vaccine (Lot No.201206204, 0.5 mL/vial) was manufactured by Liaoning Chengda biology Co., Ltd., Shenyang, China. The manufacturing process of Ormutivimab and the excipient meet the requirements of the Good Manufacturing Practice of Medical Products (GMP). HRIG and vaccine are marketed products.

Drug randomization and blinding were performed by an unblinded statistician, and the drugs were packaged by those unrelated to the study. Randomized numbers were generated by SAS^®^ 9.13 (SAS Institute, Cary, NC, USA).

Unilateral or bilateral lateral thigh muscles were selected for multi-point injection of a single Ormutivimab, HRIG, or placebo dose on Day 0. The rabies vaccine was injected in Essen’s regimen on Days 0, 3, 7, 14, and 28.

### 2.3. Pharmacodynamic Indexes

Venous blood was sampled before drug injection (D0) and on Days 3, 7, 14, 28 and 42, or the terminated day. Serum samples were then prepared for measuring the level of rabies virus neutralizing antibody (pharmacodynamic indexes). The level of neutralizing antibody was measured with a validated rapid fluorescent focus inhibition test (RFFIT) [9] at the National Institute for Food and Drug Control, Beijing, China. For RFFIT, CVS-11 was used as the challenge virus, BSR cells as the adapted cells, and WHO rabies immunoglobulin (WHO STD) as the reference serum.

### 2.4. Pharmacodynamic Models

A nonlinear mixed effects method was used for modeling. The pharmacodynamic characteristics of the drugs in the subjects were established, and their inter- and intra-individual variations were described. The factors affecting the characteristics of efficacy were analyzed [10].

All the parameters of the model were estimated using the first-order conditional estimation using the interaction (FOCEI) method [11]. The two-compartment model was used in the modeling of the phase IIa data. The E_max_ model was used in the modeling of the data from the placebo group in the phase IIb study, and the direct link model was used in the modeling of the data from the other groups in the phase IIb study.

### 2.5. Random Effects Model and Fixed Effects Model

The two commonly used models in such studies are the random effects model (REM) and fixed effects model (FEM) [12].

REM was used to describe inter- and intra-individual variations. The exponential model was used for inter-individual variation:

P_i_ = P_TV_ × exp(η_i_) (1)

P_TV_ was the population mean. P_i_ was the estimated value in the individual, and η_i_ was the inter-individual variation due to random effects, which was assumed to be normally distributed, with mean 0 and variance ω^2^.

The additive error model, proportional error model, and mixed error model were used for intra-individual (residual) variation.

Additive error model:

Y_obs,ij_ = Y_pred,ij_ + ε_ij,2_ (2)

Proportional error model:

Y_obs,ij_ = Y_pred,ij_ × (1 + ε_ij,1_) (3)

Mixed error model:

Y_obs,ij_ = Y_pred,ij_ × (1 + ε_ij,1_) + ε_ij,2_ (4)

Y_obs,ij_ was the observed value of the drug concentration in the blood, or efficacy. Y_pred,ij_ was the predicted value of the drug concentration in the blood, or efficacy. ε_ij,1_ was the proportional intra-individual variation. ε_ij,2_ was the additive intra-individual variation. ε_ij,1_ and ε_ij,2_ were assumed to be normally distributed, with mean 0 and variance σ_ij,1_^2^ and σ_ij,2_^2^.

FEM was used to add variables to the basic model to modify the parameters and to observe the impact of such variables on the model. The forward and backward selection method was used to screen the variables, with their clinical significance taken into consideration. In the forward selection, if the introduction of a variable into the model resulted in the difference between the original function value of the basic model and the new function value Δ > 3.84, the model was considered to be significantly improved by the addition of this variable (*p* < 0.01). In the backward selection, if the removal of a variable from the model resulted in the difference between the original function value and the new function value Δ < 6.63, the model was considered to be significantly improved with the addition of this variable (*p* < 0.01). Continuous covariates were introduced by the linear model or the exponential model.

Linear model:

P_TV_ = θ_1_ × [1 + θ_2_ × (COV − COV_median_)] (5)

Exponential model:

P_TV_ = θ_1_ × (COV/COV_median_)^θ_2_^ (6)

θ_1_ was the population mean when the median of the individual covariate and the population covariate were equal, θ_2_ describes the relationship between the population mean and the covariate, and COV was the individual covariate.

Categorical covariates were introduced using the following model:

(7) $$P_{\mathrm{TV}}=\{\begin{array}{cc} \theta_{1} & \mathrm{if}\;\mathrm{cov}=0\\ \theta_{1}{+\theta }_{2} & \mathrm{if}\;\mathrm{cov}=1 \end{array}$$

θ_1_ is the population mean when the covariate is 0, θ_2_ represents the relationship between the population mean and the covariate when the covariate is 1, and cov is the value of the covariate.

### 2.6. Analysis of Covariance

After determining the basic model, the covariates, such as gender, age, height, weight, BMI, genotype, and solvent for antibody preparation, were introduced. If *p* < 0.01 (the objective function value decreased by 6.63 in the chi-square distribution corresponding to one degree of freedom, *p* = 0.01), the model was considered significantly improved by the addition of this covariate. Such covariates were used for the subsequent screening.

The significant covariates were introduced into the basic model sequentially, with the covariate causing the largest absolute value change of the objective function introduced first. If *p* < 0.01, the covariate was retained in the model, and then the next variable was introduced. This step was repeated until no further significant variable could be introduced, and thus a full regression model (FRM) was established.

Following the establishment of FRM, the fixed effect factors were removed from the FRM one by one for necessity analysis. If the value of *p* was less than 0.005, the variable was significant and retained in the model; otherwise, it would be removed. After the evaluation of all the fixed effect factors and removal of the factors as needed, a final model was established.

### 2.7. Model Evaluation Method

The goodness-of-fit (GOF) test was used to evaluate the final model [13,14]. The stability of the model was evaluated using the bootstrap method [15]. The original data were sampled 1000 times to form 1000 new datasets, the model parameters of which were calculated. The nonparametric method was used to calculate the 95% confidence interval of the parameters of the datasets [16].

### 2.8. Software for Analysis

The analysis was performed using NONMEM software (Version 7.3, ICON Development Solutions, Ellicott City, ML, USA).

The interface used was Wings for NONMEM (Version 751, University of Auckland, Auckland, New Zealand). The screening of covariates and modeling were performed using Perl Speaks NONMEM (Version 3.2.4, Uppsala University, Uppsala, Sweden). The data plotting software was R package Xpose (Version 4.0, Uppsala University, Uppsala, Sweden) [17].

## 3. Results

### 3.1. Study Population

A total of 60 subjects (27 males and 33 females) were enrolled in phase IIa, with an average age of 37.7 years and an average body weight of 68.1 kg (Table 1). A total of 240 subjects (108 males and 132 females) were enrolled in phase IIb, with an average age of 39.7 years and an average body weight of 64.7 kg (Table 2). The blood pressure and heart rate of all subjects met the inclusion criteria of the trial.

### 3.2. Data Review

**Figure 1:**
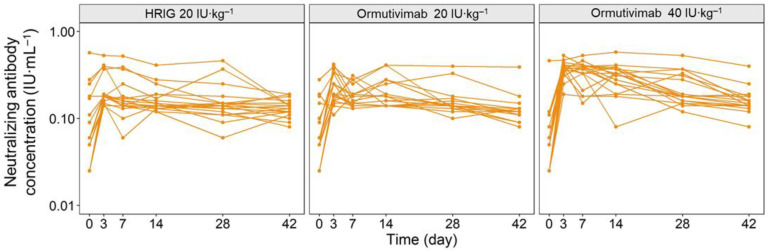
Concentration–time curve of phase IIa.

**Figure 2:**
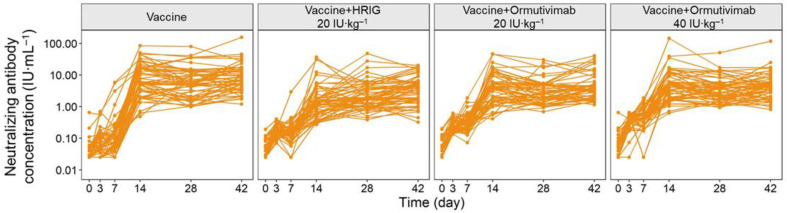
Concentration–time curve of phase IIb.

The concentration–time curves of phase IIa and phase IIb demonstrated clear linear pharmacodynamic characteristics (Figure 1 and Figure 2). The level of neutralizing antibodies increased within 0 to 3 days after the administration of Ormutivimab or HRIG in phase IIa, indicating the passive increase of neutralizing antibodies in the blood caused by Ormutivimab or HRIG injection. The level of neutralizing antibodies elevated with the increase in the dose of Ormutivimab or HRIG. After 3 days, the concentration of the neutralizing antibodies was maintained at a certain level and then decreased slowly, resulting in a relatively gentle concentration–time curve. In addition, the concentration of the neutralizing antibodies remained at a low level (<1 IU·mL^−1^). Similar to the results in phase IIa, an initial increase in neutralizing antibody level was observed in phase IIb within 0 to 3 days, suggesting the passive immunity caused by Ormutivimab or HRIG injection. However, unlike the results in phase IIa, a subsequent significant increase in the neutralizing antibody level was found from day 7 to day 14, which then leveled off to a flat plateau.

The subjects in the phase IIa trial were randomly assigned to 3 groups, with 20 subjects in each group. The subjects in each group received Ormutivimab (20 IU·kg^−1^) only, Ormutivimab (40 IU·kg^−1^) only, or HRIG (20 IU·kg^−1^) only, respectively.

The subjects in the phase IIb trial were randomly assigned to 4 groups, with 60 subjects in each group. The subjects in each group received Ormutivimab (20 IU·kg^−1^), Ormutivimab (40 IU·kg^−1^), HRIG (20 IU·kg^−1^), or placebo, respectively, in combination with rabies vaccine.

### 3.3. Basic Model Selection

**Figure 3:**
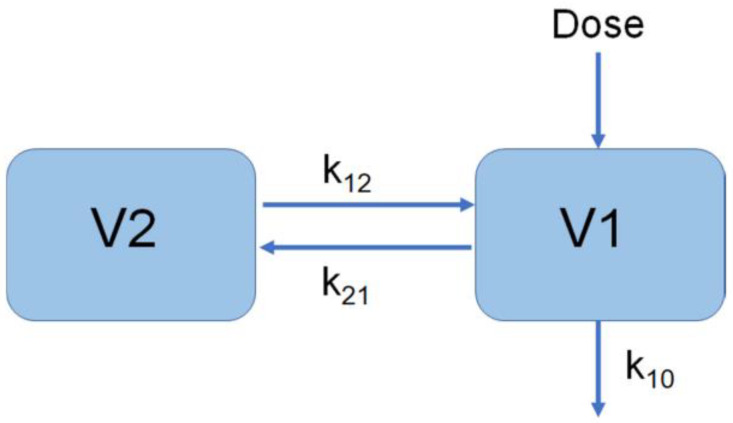
Schematic diagram of phase IIa model.

The two-compartment model was selected for modeling in phase IIa (Figure 3) [18].

Dose is the drug administration, V_1_ is the central chamber distribution volume, V_2_ is the peripheral chamber distribution volume, K_10_ is the elimination rate constant, and K_12_ and K_21_ are the transport rate constants.

(8) $$\begin{array}{l} \kappa_{10}=\frac{{\mathrm{CL}}_{1}}{V_{1}}\\ \kappa_{12}=\frac{{\mathrm{CL}}_{2}}{V_{1}}\\ \kappa_{21}=\frac{{\mathrm{CL}}_{2}}{V_{2}}\\ {A=\kappa }_{10}{+\kappa }_{12}{+\kappa }_{21} \end{array}$$

(9) $$\begin{array}{l} L_{1}=\frac{A+\sqrt{A^{2}-4\times \kappa_{10}\times \kappa_{21}}}{2}\\ L_{2}=\frac{A+\sqrt{A^{2}-\;4\;\times \kappa_{10}\times \kappa_{21}}}{2}\\ C_{1}=\frac{{(\kappa }_{21}-L_{1})\times V_{1}}{L_{2}-L_{1}}\\ C_{2}=\frac{{(\kappa }_{21}-L_{2})\times V_{1}}{L_{1}-L_{2}}\\ {\mathrm{TY}}_{1}=\mathrm{Dose}\times K_{a}\times \frac{C_{1}{(e}^{-L_{1}\times \mathrm{Time}}-e^{-K_{a}\times \mathrm{Time}})}{K_{a}-L_{1}}\\ {\mathrm{TY}}_{2}=\mathrm{Dose}\times {\;K}_{a}\times \frac{C_{2}{(e}^{-L_{2}\times \mathrm{Time}}-e^{-K_{a}\times \mathrm{Time}})}{K_{a}-L_{2}}\\ Y_{1}{=\mathrm{TY}}_{1}{+\mathrm{TY}}_{2}{+C}_{0} \end{array}$$

Dose referred to the administered dose of the drug, V_1_ was the central volume of distribution, V_2_ was the peripheral volume, K_10_ was the elimination rate constant, K_12_ and K_21_ were the transport rate constants, and C_0_ was the concentration of neutralizing antibodies in vivo without drug administration.

**Figure 4:**
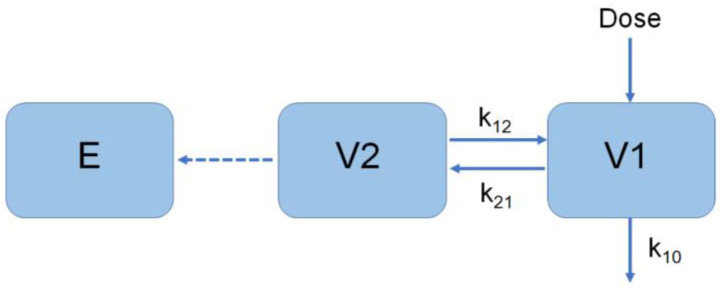
Schematic diagram of phase IIb model.

The E_max_ model was used for the modeling of the placebo group in phase IIb. Both the direct link model and the piecewise superposition model were considered for the modeling of the other groups in phase IIb [19]. The direct link model was adopted, based on curve fitting results (Figure 4):

Dose is the drug administration, V1 is the central chamber distribution volume, V2 is the peripheral chamber distribution volume, K10 is the elimination rate constant, and K_12_ and K_21_ are the transport rate constants. E_max_ uses a time-dependent Emax model, and E is the effect (neutralizing antibody activity).

(10) $$\begin{array}{l} Y_{2}{=E}_{0}+\frac{E_{\max}\times {\mathrm{Time}}^{\gamma }}{{\mathrm{ET}}_{50}{}^{\gamma }+{\mathrm{Time}}^{\gamma }}\\ {E=Y}_{1}{+Y}_{2} \end{array}$$

A time-dependent E_max_ model was utilized, with E as the effect (the activity of neutralizing antibodies).

### 3.4. Covariate Screening

Covariates were screened one by one using the stepwise method, described earlier in this article. The screening process of the covariant model, the correlation diagram of the parameters and covariates of the model, and the physiological significance of the covariates were taken into consideration during the screening. The key covariates analyzed included antibody type, body weight, and gender. Finally, antibody type was determined as the covariate significantly affecting the model.

(11) $$\begin{array}{l} E_{\max}{=E}_{\max\_\mathrm{HRIG}}+\{\begin{array}{cc} 0 & \mathrm{HRIG}\\ 0.143 & \mathrm{rHRIG} \end{array}\\ {\mathrm{ET}}_{50}{=\mathrm{ET}}_{50\_\mathrm{HRIG}}+\{\begin{array}{cc} 0 & \mathrm{HRIG}\\ -3.8 & \mathrm{rHRIG} \end{array} \end{array}$$

### 3.5. Final Model and Its Evaluation

The pharmacodynamic parameters were obtained by the final model (Table 3). The original data were re-sampled, using the bootstrap approach, 1000 times. The 1000 new datasets were fitted, 873 of which fitted successfully (87.3%). The median of the bootstrap approaches was approximate to that of the parameter estimates; the confidence interval of the bootstrap approaches coincided with that of the parameter estimates. The 95% confidence interval of the fixed effects parameter group did not include 0, indicating that the stability and reliability of parameter estimation were not significantly affected by the sample distribution. The mean values of all distributions were similar to the median, and the model showed good stability.

### 3.6. Evaluation of the Final Model

**Figure 5:**
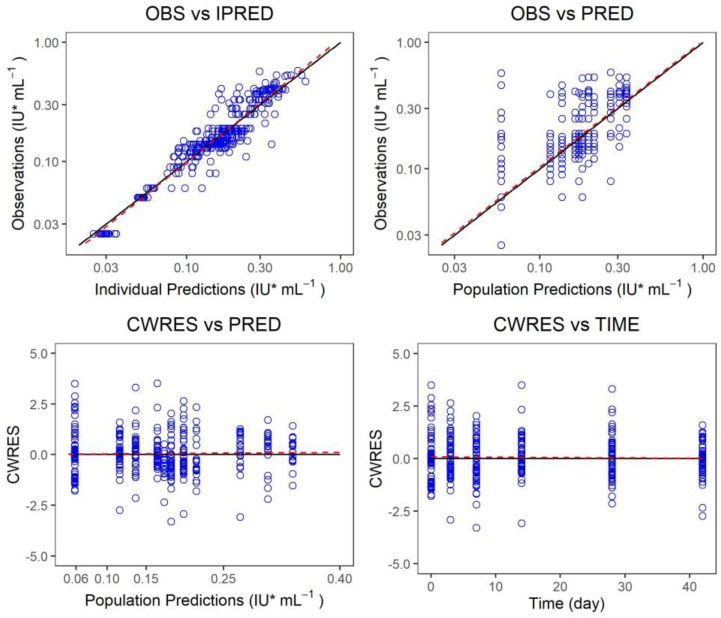
Fitting graph of the phase IIa model.

**Figure 6:**
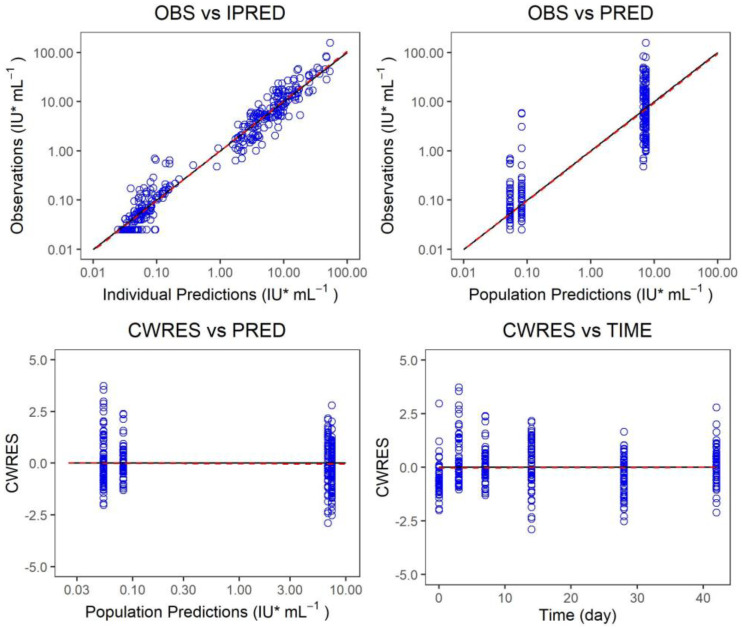
Fitting graph of the phase IIb model.

**Figure 7:**
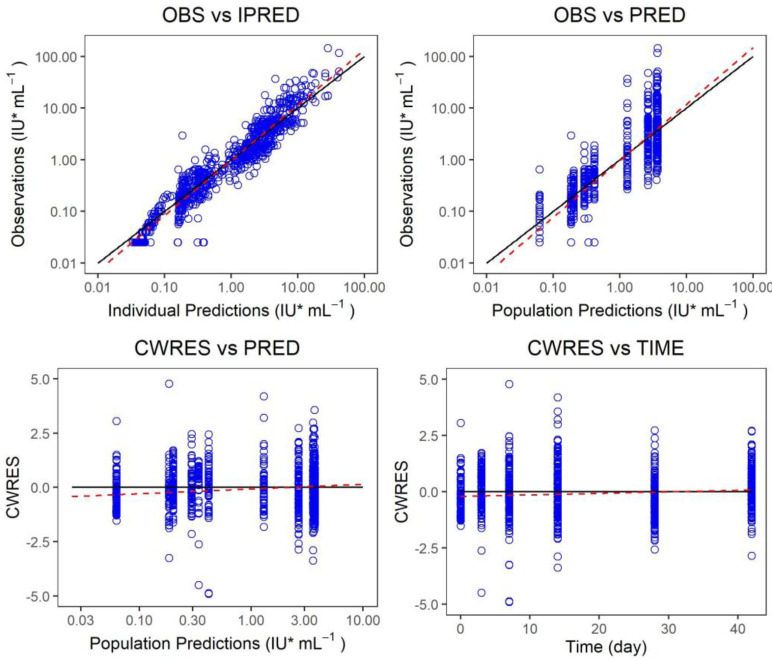
Fitting graph of the final model.

The measured value of the final model was plotted versus the predicted value of the group or individuals, and the conditional weighted residual was plotted versus the predicted value of the group and independent variables, and the model fitting was evaluated (Figure 5, Figure 6 and Figure 7). There was a strong correlation between the measured value and the predicted group value or the predicted individual value. The standard line nearly coincided with the regression line. Most of the conditional weighted residual errors (CWRES) were between ±4, and they were evenly distributed on both sides of the coordinate axis. The model successfully described the change trend of the neutralizing antibody concentration over time in healthy adult subjects after the administration of Ormutivimab + vaccine or HRIG + vaccine.

The measured value of the phase IIa model was plotted versus the predicted value of the group or individuals. The conditional weighted residual was plotted versus the predicted value of the group and the independent variables. The model fitting was evaluated. The black line shows the standard line, and the red line shows the regression line in the graph.

The measured value of the phase IIb model was plotted versus the predicted value of the group or individuals. The conditional weighted residual was plotted versus the predicted value of the group and independent variables. The model fitting was evaluated. The black line shows the standard line, and the red line shows the regression line in the graph.

The measured value of the final model was plotted versus the predicted value of the group or individuals. The conditional weighted residual was plotted versus the predicted value of the group and independent variables. The model fitting was evaluated. The black line shows the standard line, and the red line shows the regression line in the graph.

### 3.7. Simulation Results of Different Scenarios Using the Final Model

**Figure 8:**
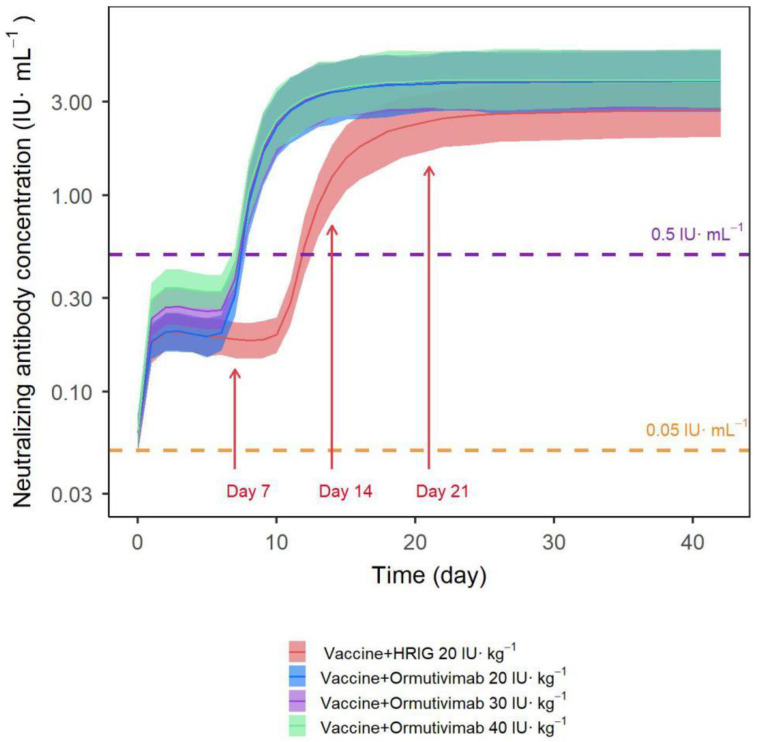
Drug concentration–time curve and 95% confidence interval of different administration regimens.

The final model was used to set up the simulation in different scenarios, resulting in the concentration–time curve and the 95% confidence interval of different dosing regimens (Figure 8), including simulated Ormutivimab (30 IU·kg^−1^) + vaccine. As shown in the figure, the levels of neutralizing antibodies were comparable between the Ormutivimab 20 IU·kg^−1^ + vaccine group and the HRIG 20 IU·kg^−1^ + vaccine group within 7 days after administration. We found that the Ormutivimab 40 IU·kg^−1^ + vaccine group had a higher level of neutralizing antibodies in the same time frame. By analyzing the concentration–time curves, we found that the vaccine induced the production of effective antibodies at around 7–10 days after vaccination. The levels of neutralizing antibodies in all groups reached the effective protective level (>0.5 IU·mL^−1^). Although the levels of neutralizing antibodies reached the peak in all groups by day 21 after administration, the Ormutivimab + vaccine group peaked higher and faster than the HRIG + vaccine group. After peaking, the Ormutivimab (20 IU·kg^−1^) + vaccine group, the Ormutivimab (40 IU·kg^−1^) + vaccine group, and the HRIG + vaccine group showed similar levels of antibody neutralization.

## 4. Discussion

This is the first report of PPD modeling of Ormutivimab in healthy adult subjects. The total number of participants was 300, including 60 in phase IIa and 240 in phase IIb. We used PPD modeling to compare the activity, reaction characteristics, and impacting factors of the neutralizing antibodies induced by the co-administration of Ormutivimab or HRIG with a purified Vero cell rabies vaccine, so as to recommend the target dose of Ormutivimab in a phase III study.

The REM and FEM were used to establish the final model. The REM incorporated the inter-individual variations into the final model, whereas the FEM incorporated the inter-group variations into the final model. Covariates were screened, one by one, using a stepwise method. The screening process of the covariant model, the correlation diagram of the parameters and covariates of the model, and the physiological significance of the covariates were taken into consideration. The key covariates analyzed were antibody type, body weight, and gender, and antibody type was determined as the covariate significantly affecting the model. After screening, we obtained the final full regression model (FRM), which was then subjected to backward elimination to obtain the final covariate model.

The final model was evaluated using the GOF test. The bootstrap method was used to re-sample the original data 1000 times, and 873 of the 1000 datasets (87.3%) were fitted successfully. Therefore, the final model could simulate clinical data with high accuracy. The median of the bootstrap parameter estimates was close to that of the parameter estimates. The confidence interval of the bootstrap parameter estimates coincided with that of the parameter estimates. The 95% confidence interval of the fixed effects parameter group did not include 0, indicating that the stability and reliability of parameter estimation were not significantly affected by the sample distribution. The mean values of all distributions were similar to the median, and the model exhibited good stability. We concluded that the model could successfully describe the change trend of the neutralizing antibody concentration over time in healthy adult subjects after administration of Ormutivimab + vaccine or HRIG + vaccine.

We used the final model to set up simulations in different scenarios and generated the concentration–time curve and 95% confidence interval of different dosing regimens. By analyzing the concentration–time curves, we found that the Ormutivimab (20–40 IU·kg^−1^) + vaccine group demonstrated clear linear pharmacodynamic characteristics within 7 days after administration. This was before the body was able to produce effective antibodies induced by the vaccine. The levels of neutralizing antibodies were comparable between the Ormutivimab 20 IU·kg^−1^ + vaccine group and the HRIG 20 IU·kg^−1^ + vaccine group in the same time frame. The passive immunity during this period could enable immediate neutralization of the rabies virus without a host immune response. The goal of RIG administration is to achieve a high local concentration of neutralizing antibodies via local infiltration into and around the site of exposure, thus blocking the spread of the virus before the vaccine-induced production of potent antibodies by the body [1]. The effect of passive immunity is rapid, but transient, while active immunity can last for years, although the rabies vaccine requires 1–2 weeks to induce the production of IgG antibodies [20]. In addition, the increased level of neutralizing antibodies in the peripheral circulation results mainly from vaccine-induced active immune response. According to the WHO position paper on rabies vaccines, the PEP regimen, whether including passive immunizing agents or not, should achieve a serum rabies neutralizing antibody titer equal to or greater than 0.5 IU·mL^−1^ at 7–14 days after vaccination, in order to provide effective protection [21]. By analyzing the concentration–time curves, we found that the vaccine induced the production of effective antibodies at around 7–10 days after vaccination. The levels of neutralizing antibodies in all groups reached the effective protective level (>0.5 IU·mL^−1^). Moreover, the Ormutivimab + vaccine group can reach the effective protection level (0.5 IU·mL^−1^) earlier than the HRIG 20 IU·kg^−1^ + vaccine group. The administration of Ormutivimab (20–40 IU·kg^−^^1^) did not negatively affect the protective effect of the vaccine. Although the levels of neutralizing antibodies reached the peak in all groups by day 21 after administration, the Ormutivimab + vaccine group peaked higher and faster than the HRIG + vaccine group, and the overall neutralizing antibody level was slightly higher. Furthermore, a fifth immunization at Day 28 did not result in an increased level of neutralizing antibodies, suggesting that Ormutivimab (20–40 IU·kg^−^^1^) did not interfere with the final immune response induced by the vaccine. The Ib phase study (being sorted out) also presented similar results to those listed above. Compared with HRIG (20 IU·kg^−1^), Ormutivimab (20–40 IU·kg^−1^) may have less impact on the active immunization of the vaccine. Summarizing the published articles [5,22,23], we found that rhRIG from different manufacturers had different effects on the active immunization of the vaccine because of the different surface sites of the rabies virus neutralized by antibodies and different dosages.

This study has several limitations. First, since the participants in this trial are healthy adults, we can only recommend the appropriate dosage by simulating the pharmacodynamic indicators. Secondly, the age group selected for the trial has limitations, and young people aged < 18 and elders aged > 55 may have more chance of being exposed to dogs and other infected animals. Therefore, people with rabies exposures and people of a wider age should be considered in further trials.

## 5. Conclusions

In the phase II study, we also conducted a safety assessment. In phase IIa, the incidence of adverse reactions was the same in the Ormutivimab (20 IU·kg^−1^) group, Ormutivimab (40 IU·kg^−1^) group, and HRIG (20 IU·kg^−1^) group, all of which were 20%. In phase IIb, the incidence of adverse events in the Ormutivimab (20 IU·kg^−1^) + vaccine group (17.2%) was lower than that in the Ormutivimab (40 IU·kg^−1^) + vaccine group (36.7%) and HRIG (20 IU·kg^−1^) + vaccine group (40.3%).

According to our research results and the simulation results of the final model, 20 IU·kg^−1^ is recommended as the target dose of Ormutivimab in the confirmatory study.

## Figures and Tables

**Table 1:** Demographics characteristics of phase IIa clinical trial (mean ± SD).

	HRIG 20 IU·kg^−1^	Ormutivimab 20 IU·kg^−1^	Ormutivimab 40 IU·kg^−1^	Total
Subjects	20	20	20	60
Age (year)	39.96 ± 8.43	35.05 ± 9.23	38.17 ± 9.18	37.73 ± 9.04
Body Weight (kg)	67.15 ± 12.18	70.40 ± 12.27	66.75 ± 12.63	68.10 ± 12.26
SBP (mmHg)	118.4 ± 8.1	120.6 ± 9.6	118.4 ± 10.7	119.1 ± 9.4
DBP (mmHg)	78.7 ± 6.9	80.5 ± 8.7	77.3 ± 7.0	78.8 ± 7.5
Heart rate (bpm)	70.6 ± 9.2	75.2 ± 9.6	69.4 ± 6.4	71.7 ± 8.7
Sex (male/female)	8/12	12/8	7/13	27/33

**Table 2:** Demographics characteristics of phase IIb clinical trial (mean ± SD).

	Vaccine	HRIG 20 IU·kg^−1^ + Vaccine	Ormutivimab 20 IU·kg^−1^ + Vaccine	Ormutivimab 40 IU·kg^−1^ + Vaccine	Total
Subjects	60	62	58	60	240
Age (year)	39.64 ± 9.00	40.38 ± 7.53	39.89 ± 8.00	38.95 ± 7.90	39.72 ± 8.09
Body Weight (kg)	64.63 ± 10.35	64.65 ± 10.21	63.19 ± 10.80	66.33 ± 13.09	64.71 ± 11.15
SBP (mmHg)	114.7 ± 11.8	115.3 ± 10.2	114.8 ± 10.4	116.4 ± 13.2	115.3 ± 11.4
DBP (mmHg)	77.0 ± 9.4	77.4 ± 9.7	76.7 ± 8.1	78.3 ± 9.8	77.4 ± 9.3
Heart rate (bpm)	78.5 ± 4.8	77.9 ± 4.7	78.0 ± 5.7	78.2 ± 5.0	78.2 ± 4.9
Sex (male/female)	31/29	24/38	23/35	30/30	108/132

**Table 3:** Final model parameters.

	Final Model	Bootstrap (N = 873 *)	
	Estimates (RSE%)	Median	95% PI
PD parameter			
*E_max_*, IU/mL	3.6 (15.1)	3.6	(3.17, 4.46)
θ_1_	0.143 (40.1)	0.146	(0.049, 0.260)
ET_50_, day	10.5 (8.4)	10.4	(9.46, 11.6)
θ_2_	−3.8 (19.1)	−3.72	(−4.90, −2.86)
Gamma	7.66 (22.6)	7.68	(5.81, 13.3)
E_0_	−3.19 (16.9)	−3.19	(−4.08, −2.79)
Inter-individual variability			
ω (*E_max_*), %	9.0 (16.8)	8.9	(6.7, 10.4)
ω (Gamma), %	56.1 (22.7)	57.4	(34.4, 80.1)
Residual Error			
σ (additive)	0.245 (3.8)	0.241	(0.207, 0.268)
σ (proportional), %	9.4 (53.9)	10.3	(2.0, 21.8)

* Number of successful times calculated by 1000 bootstrap samplings.

## Data Availability

Not applicable.
